# Invasive Fresh Water Snail, China

**DOI:** 10.3201/eid1307.061360

**Published:** 2007-07

**Authors:** Qiao-Ping Wang, Xiao-Guang Chen, Zhao-Rong Lun

**Affiliations:** * Sun Yat-Sen (Zhongshan) University, Guangzhou, People’s Republic of China; † Southern Medical University, Guangzhou, People’s Republic of China

**Keywords:** Pomacea canaliculata, angiostrongyliasis, distribution, China, snail, letter

**To the Editor:** *Pomacea canaliculata*, an invasive freshwater snail native to South America, was first introduced as a food to Taiwan in1979 and then to Mainland China in 1981 (*1*). It adapted well to the environment, particularly to the southern parts of the Mainland, spreading rapidly to more than 10 provinces (Figure) and causing tremendous damage to agriculture and the ecosystem (*1*,*2*). Thousands of hectares of rice, vegetables, and other crops in these provinces were destroyed (*2*).

**Figure:**
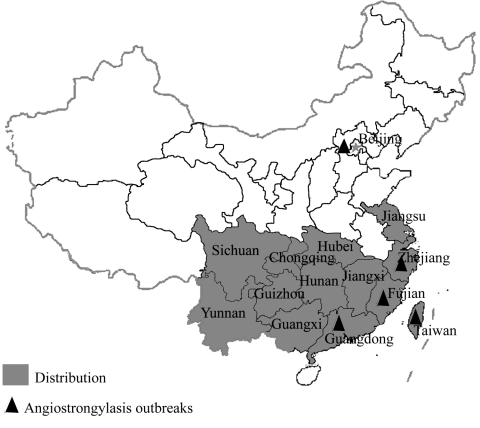
Distribution of *Pomacea canaliculata* in China. The dark triangles indicate the regions where angiostrongyliasis outbreaks were reported due to ingestion of raw or undercooked *P. canaliculata* snails.

Even more alarming were the multiple outbreaks of a severe brain disease (angiostrongyliasis) in Taiwan that were linked to *P. canaliculata (3,4).* Angiostrongyliasis is caused by *Angiostrongylus cantonensis*, a lung nematode of wild rodents, commonly known as the rat lungworm. In Mainland China, epidemiologic evidence also indicates that *P. canaliculata,* because of its high susceptibility to *A. cantonensis,* is becoming the most important natural intermediate host for this parasite (*5*). Previously, other terrestrial snails like *Achatina fulicia,* and some species of slugs such as *Philomycus bilineatus* were regarded as the major intermediate hosts for *A. cantonensis* (*6*). Epidemiologic survey results from 1997 to 1999 demonstrated that 20.8%–69.4% of *P. canaliculata* were infected with *A. cantonensis* in some regions of Guangdong, Zhejiang, and Fujian Provinces (*5*). Even in provinces where the snail is not found, a high incidence and prevalence of infection occur because of its widespread distribution, high susceptibility to *A. cantonensis*, and growing popularity as a food. In 1997, 2002, and 2002, ingestion of raw or undercooked *P. canaliculata* meat led to 3 outbreaks of angiostrongyliasis infecting >100 patients (*6**,**7*). A 2006 outbreak in Beijing infected 131 persons (*8*). Based on the biologic characteristics of *P. canaliculata*, blocking its life cycle is one of the most effective methods to limit the outbreak of angiostrongyliasis. However, the current widespread distribution of *P. canaliculata* in China and the lack of a highly effective control method make the disease extremely difficult to eliminate (*9*). More outbreaks associated with ingestion of this snail will likely occur if food safety rules are not strictly enforced. Citizens must also be educated to avoid eating raw, undercooked snail meat or raw vegetables from regions that may be contaminated with infective mucous trails deposited by these snails (*10*).

## References

[1] Xu HG, Qiang S. Inventory invasive alien species in China. Beijing: Chinese Environmental Science Press; 2004.

[2] Zhou XC. Risk analysis of invasive *Pomacea canaliculata.* Jian Yan Jian Yi Ke Xue. 2004;14:37–9.

[3] Tsai HC, Liu YC, Kunin CM, Lai PH, Lee SS, Chen YS, Eosinophilic meningitis caused by *Angiostrongylus cantonensis* associated with eating raw snails: correlation of brain magnetic resonance imaging scans with clinical findings. Am J Trop Med Hyg. 2003;68:281–5. 12685630

[4] Tsai HC, Liu YC, Kunin CM, Lee SS, Chen YS, Lin HH, Eosinophilic meningitis caused by *Angiostrongylus cantonensis*: report of 17 cases. Am J Med. 2001;111:109–14. 10.1016/S0002-9343(01)00766-5 11498063

[5] Lin W, Wang XT. Epidemiological survey of angiostrongyliasis in Mainland China. Chin J Zoonoses. 2004;20:1004–5.

[6] Hollingsworth RG, Cowie RH. Apple snails as disease vectors. In: Joshi RC, Sebastian LC. Global advances in ecology and management of gloden apple snails. Nueva Ecija: Philippine Rice Institute; 2006. p. 121–32

[7] Chen XG, Li H, Lun ZR. Angiostrongyliasis, mainland China. Emerg Infect Dis. 2005;11:1645–7. 16355510 10.3201/eid1110.041338 PMC3366736

[8] The number of the case of angiostrongyliasis caused by eating *P. canaliculata* reached 131 in Beijing. [cited 2007 Jan 21]. Available from http://news3.xinhuanet.com/fortune//2006-09/12/content_5078790.htm

[9] Cowie RH. Apple snails (Ampullariidae) as agricultural pests: their biology, impacts and management. In: Barker GM. Molluscus as crop pests. Wallingford (UK): CABI Publishing; 2002. p. 145–92

[10] Tsai HC, Lee SS, Huang CK, Yen CM, Chen ER, Liu YC. Outbreak of eosinophilic meningitis associated with drinking raw vegetable juice in southern Taiwan. Am J Trop Med Hyg. 2004;71:222–6. 15306715

